# Real-time vectorcardiography simulator system

**DOI:** 10.1371/journal.pone.0345612

**Published:** 2026-03-31

**Authors:** William R. Rodriguez, Hernan A. Bernal, Diana Patricia Amador-Munoz

**Affiliations:** 1 Department of Electronic Engineering, Faculty of Engineering, Pontificia Universidad Javeriana, Bogota, Colombia; 2 School of Medicine and Health Sciences, biomedical engineering. Universidad del Rosario, Bogota, Colombia; 3 School of Medicine and Health Sciences, Neurovitae research Center, Universidad del Rosario, Bogota, Colombia; Polytechnic University of Marche: Universita Politecnica delle Marche, ITALY

## Abstract

Vectorcardiography provides a spatial orientation and magnitude of the electrical activity of the heart, but its complexity and lack of interactive resources have limited its application in medical and bioengineering education. To bridge this pedagogical gap, we developed a low-cost, real-time vectorcardiography simulator integrating a physical wet-lab interface and digital signal processing. The hardware setup consists of a conductive medium and electrodes, enabling users to manually simulate a cardiac vector and observe the resultant electrophysiological signal consequences in real time. The system was validated through three complementary methods: theoretical conformity analysis, emulation of real ECG data from database, and user-driven waveform generation tests. A pilot study with medical students and instructors provided empirical evidence of the educational value of the device, indicating that the active, hands-on nature of the system might foster deeper cognitive engagement and facilitates the integration of complex electrophysiological concepts. By providing open-source software and cost-effective hardware, this simulator offers a scalable solution to enhance cardiac electrophysiology education and promote the broader adoption of VCG in clinical practice.

## Introduction

### Context and problem

Vectorcardiography (VCG) provides a comprehensive graphical representation of cardiac electrical activity, offering insights into the direction and magnitude of the electrical vectors of the heart [1]. This diagnostic modality can reveal pathologies such as myocardial ischemia and ventricular hypertrophy that may be ambiguous in standard scalar electrocardiograms (ECG) [2]. Despite its clinical potential, VCG remains underutilized, primarily due to the inherent complexity of interpreting its abstract 2D or 3D dimensional vector loops, which present a significant learning curve for medical and bioengineering students [3,4]. They often struggle to effectively correlate the temporal waveforms of a conventional bipolar or 12-lead ECG with the intricate spatial dynamics of the heart vector [5].

The educational landscape in cardiac electrophysiology often faces a critical pedagogical gap. Traditional didactic approaches, while foundational, frequently fall short in fostering the deep understanding required for complex, multi-dimensional physiological phenomena like VCG [6,7]. Effective learning, particularly in biomedical sciences, benefits immensely from active and experiential methodologies, where students can engage directly with the subject matter instead of passively receiving information [5]. The challenges in mastering ECG interpretation are well-documented [8], and VCG, with its added spatial dimension, poses an even greater interpretive hurdle. Educational tools that enable active learning and hands-on experience are crucial for bridging this gap, given that they significantly enhance knowledge acquisition and retention of cardiovascular physiology [9]. Studies evaluating VCG applications in education indicate that students perceive significant benefits in understanding the relationship between ECG signals and cardiac electrical activity, and express strong interest in the utility of the VCG [4]. However, there is a notable scarcity of physical, interactive, or haptic tools specifically designed for teaching VCG principles. Current methods often require specialized and costly equipment, leading to limited accessibility and insufficient hands-on educational resources [3,4]. This lack of physically interactive platforms hinders a comprehensive understanding of how the electrical vector of the heart movement within the thorax generates the diverse ECG waveforms across various lead configurations, thereby impeding a broader VCG adoption in both clinical and educational settings. The need for tangible and manipulable models to grasp the complex electrical activity of the heart is evident in educational research, where physical models and simulations are shown to improve comprehension [10,11].

### State of the art

Current educational tools for VCG and related cardiac electrical activity can be categorized into three main types: software-based systems, static hardware systems, and mixed hardware-software systems. Software-based solutions, such as BRAVEHEART, a Matlab package proposed by Stanbeneau et al., offer algorithms for filtering, beat detection, feature extraction, and visualization of VCG loops from pre-existing digital ECG file formats [12]. Similarly, Šljivo et al. developed a multimedia educational application using Python and data from the PhysioNet database. This application allows students to visualize VCG and its relationship with ECG signals, as well as review the temporal changes of cardiac vectors [13,14]. While these software tools provide valuable visual insights and analytical capabilities, they predominantly rely on pre-recorded data or virtual interactions, thereby lacking the crucial physical interactivity necessary for true ‘active learning’ and intuitive comprehension of dynamic processes [5]. Simulation software for electrophysiological mapping is a powerful tool for visualization and manipulation of electrical activity, but often requires strong foundational technical knowledge to utilize effectively [11].

In terms of hardware initiatives, Jin et al. described a simple and inexpensive device to illustrate the Einthoven triangle, enabling students to physically interact with a representation of cardiac electrical activity and measure lead voltage values [15]. However, this device provides only static measurements and lacks the dynamic visualization of VCG loops that are essential for understanding the temporal evolution of the heart vector. More sophisticated mixed hardware-software systems, such as that proposed by Martinez et al., focus on developing individually corrected VCG lead systems using 3D anatomical models derived from computed tomography scans to optimize lead placement and simulate VCG signals [16]. While these systems advance the accuracy of VCG acquisition, their primary focus is on signal collection and anatomical precision rather than interactive pedagogical engagement for direct manipulation and real-time feedback. Despite these advancements, a significant gap remains in providing a direct, real-time, and physically interactive platform for learners to manipulate the heart vector and observe its immediate electrophysiological consequences.

### What we propose

We present a low-cost, real-time VCG simulator that uniquely integrates a physical wet-lab interface with digital signal processing and visualization. Diverging from prior works, our system empowers users to manually manipulate a ‘heart vector’ within a conductive medium and instantaneously observe the resultant electrocardiographic waveforms and vectorcardiographic loops. This hands-on approach directly addresses the identified pedagogical gap by fostering an intuitive and experiential understanding of cardiac projection principles. The system has been designed with an emphasis on cost-effectiveness, ensuring broad accessibility, while simultaneously maintaining inherent capacity for expandability and customization. This design philosophy aims to significantly promote the understanding and adoption of VCG technology within both academic and medical educational environments by offering an intuitive bridge between theoretical knowledge and practical, real-time interaction.

## Materials and methods

The methodological approach integrates a hybrid hardware-software architecture, enabling users to physically manipulate a simulated cardiac electrical vector within a conductive medium and observe its immediate electrophysiological consequences. This design prioritizes a direct, experiential learning paradigm, bridging the gap between abstract theoretical concepts and their tangible manifestations in both ECG waveforms and VCG loops. The functionality of the system and accuracy were assessed through a validation process, encompassing both technical performance verification against established theoretical models and real-world data, and a preliminary educational efficacy assessment to evaluate its pedagogical utility.

### Basic principles of vectorcardiography

**Fig 1:**
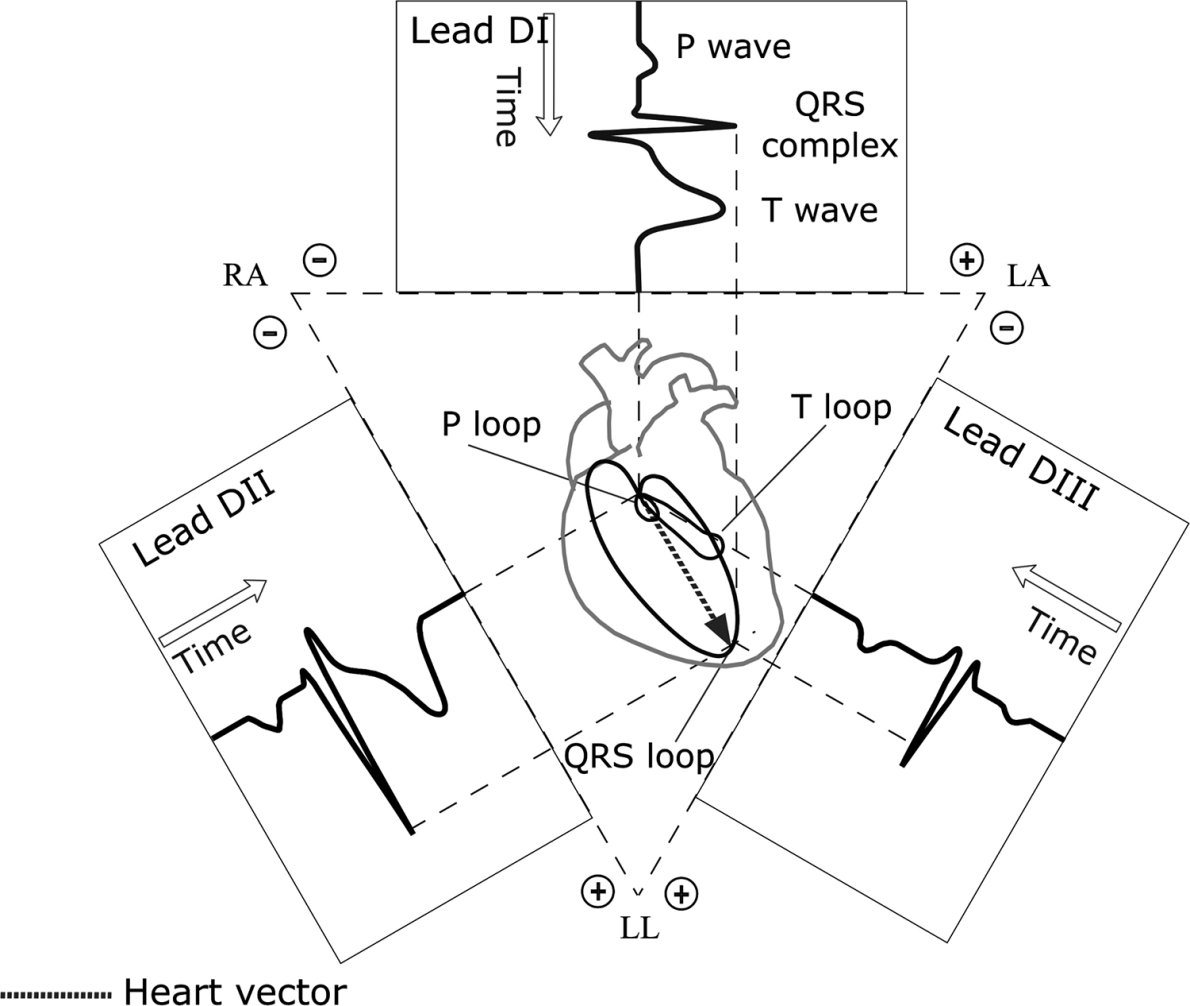
Frontal 2D representation of the heart vector. The trajectory of the instantaneous heart vector traces the frontal vectorcardiographic loops, whose projections onto the sides of the Einthoven triangle produce the corresponding electrocardiographic waveforms in Leads I, II, and **III.**

Vectorcardiography provides a dynamic 2D-3D dimensional representation of the electrical activity of the heart, complementing traditional ECG by visualizing the magnitude and spatial orientation of the cardiac electrical vector [1]. This electrical vector, representing the summation of all myocardial cellular depolarizations and repolarizations, undergoes a characteristic trajectory during each cardiac cycle [3]. Its projection onto specific planes generates the distinctive VCG loops corresponding to the P wave (atrial depolarization), QRS complex (ventricular depolarization), and T wave (ventricular repolarization) of the ECG [17–19] (see Fig. 1). Analyzing these spatial loops offers enhanced insights into cardiac abnormalities and their correlation with ECG waveforms (in Leads DI, DII, and DIII), making VCG a valuable diagnostic and educational tool [4].

However, it is important to note that VCG is an approximation of the electrical activity of the heart. Anatomical factors, such as the non-homogeneous conductivity of the human thorax and the non-central position of the heart, along with electrode placement variations, can influence the morphology and orientation of VCG loops [20]. Consequently, simulated or recorded VCG loops may differ from idealized theoretical representations. Our simulator aims to provide a tangible model for understanding these projections and their relationship to ECG signals, despite these inherent biological complexities.

### System architecture

The real-time vectorcardiography simulator is designed as a hybrid system, integrating physical hardware for biopotential generation and acquisition with specialized software for data processing, analysis, and visualization. This architecture enables users to actively manipulate a simulated cardiac electrical vector within a conductive medium, and the system simultaneously measures, processes in real-time, and displays the resulting electrical potentials as both conventional ECG waveforms and VCG loops. The design of the system prioritizes a direct, interactive experience, bridging the gap between theoretical VCG concepts and their practical manifestations.

The simulator comprises three primary interconnected modules:

1. Hardware Interaction Module: This physical interface allows for the manual generation of biopotentials within a controlled conductive environment.
2. Signal Acquisition Module: Responsible for capturing, amplifying, filtering, and digitizing the analog electrical signals from the hardware interaction module.
3. Data Processing and Visualization Module: A software-based component that receives the digitized signals, performs necessary calculations, and presents the ECG waveforms and VCG loops graphically in real-time.

**Fig 2:**
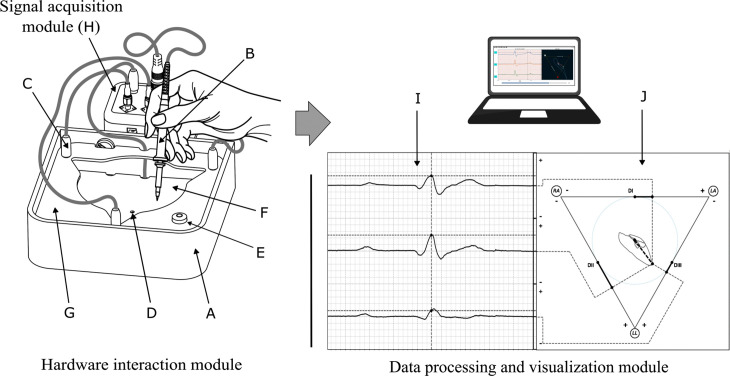
General schematic of the system. Hardware interaction module: container A, a mobile hand electrode B, fixed electrodes C, a sacrifice electrode D, a mini spirit level bubble indicator E, conductive medium (sodium chloride solution) F, and an acrylic flat piece G; Signal acquisition module H; and Data processing and visualization module with ECG waves I, and VCG loops J.

A general schematic illustrating the interaction between these modules is presented in Fig 2, and, an image of the final VCG simulator in operation is shown in operation in Fig 5.

### Hardware design and implementation

The hardware component is engineered to simulate the fundamental principles of volume conduction and biopotential measurement central to VCG. This module is segmented into the physical interaction setup and the electronic circuitry for signal acquisition.

#### Physical interaction setup.

The core of the hardware interaction module is a custom-designed, transparent container constructed from 5 mm methyl methacrylate sheets, assembled with silicone sealant. This container holds 400 mL of 0.9% sodium chloride solution, serving as a homogeneous conductive medium that mimics the bioelectrical environment of the human torso. A mini spirit level bubble indicator is integrated to ensure precise horizontal positioning, which is critical for consistent experimental conditions.

Within this container, three fixed measurement electrodes are strategically positioned at the vertices of an equilateral triangle, conceptualizing the triangle of Einthoven. These electrodes are mounted on a custom 3D-printed acrylic flat piece to maintain their geometric configuration. A mobile hand-electrode, acting as the active biopotential source, is manually manipulated by the user within the conductive medium. This mobile electrode is an adapted BNC oscilloscope probe (Input resistance: 1X 1MΩ - 10X 10MΩ; Input capacitance: 1X 85 120pF - 10X 18.5pF – 22.5pF; Bandwidth: 1X DC-6MHz - 10X DC-150MHz [21], connected via a shielded coaxial cable to minimize electrical noise. A sacrifice electrode, submerged at the bottom of the container, acts as the negative reference terminal for the circuit, crucial for defining the potential gradient. We selected the use of DC voltage (5 VDC) between the mobile and reference electrodes for its direct interpretability of lead voltages and its analogous representation of cardiac dipole phenomena [22].

To mitigate electrochemical degradation (corrosion) inherent with DC polarization in an electrolytic solution [23], the sacrifice electrode was fabricated from a combination of stainless steel and brass, selected after evaluating various metal combinations for minimal corrosion over typical usage durations. The fixed measurement electrodes, designed as female panel mount connectors (Tip Jack-Plug [24]), are connected to the signal acquisition module.

#### Signal acquisition circuitry.

The signal acquisition module is housed within a custom 3D-printed PLA nylon enclosure, mechanically coupled to the main container. This module integrates an analog front-end with a digital conversion system. It receives analog signals from the fixed measurement electrodes, the mobile hand electrode, and the sacrifice electrode.

**Fig 3:**
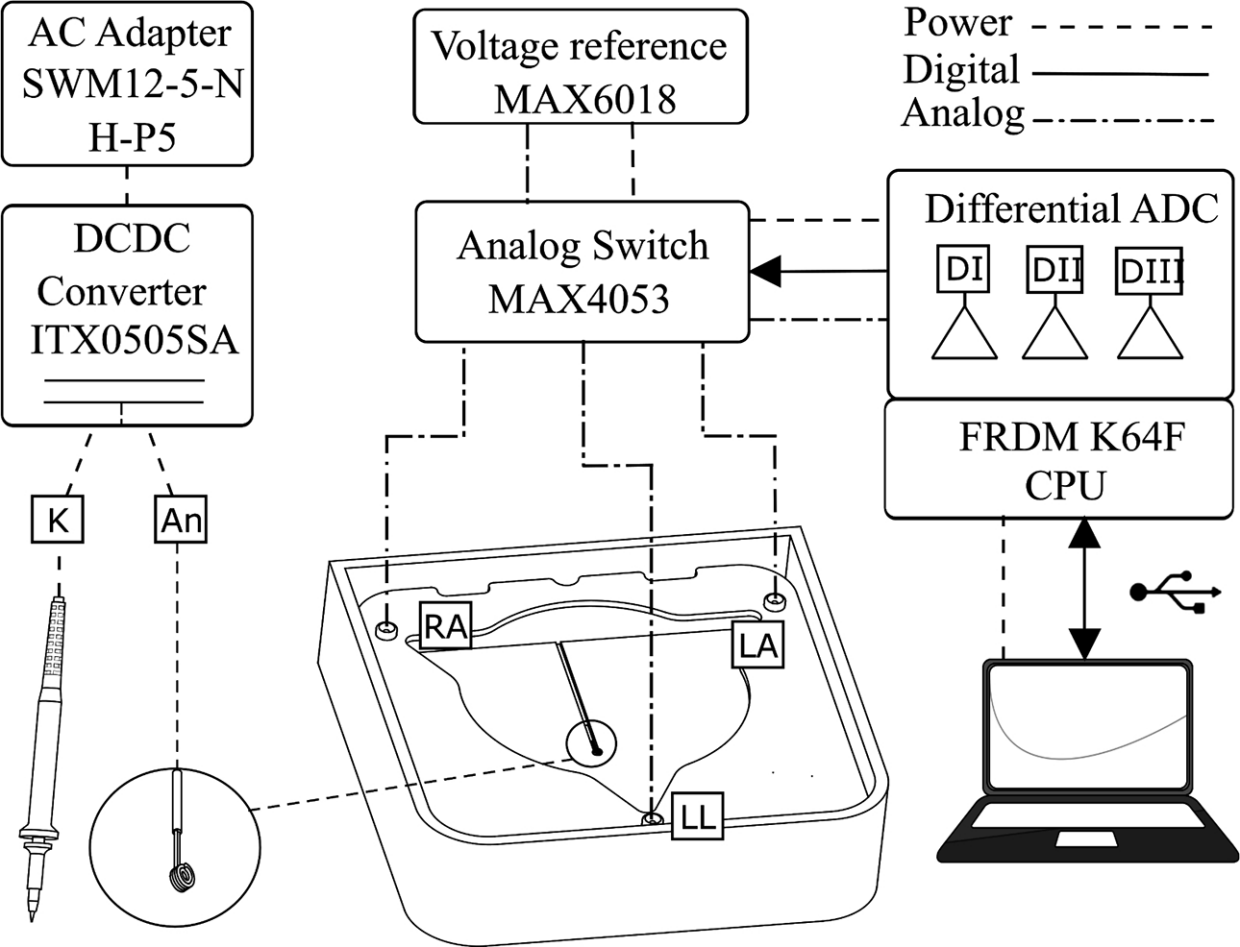
General circuitry diagram. The electronic component MAX6018 provides a stable voltage reference for the 16-bit differential ADC in the K64F microcontroller. A MAX4053 multiplexer measures each channel sequentially. System power isolation uses an ITX0505SA DC-DC converter connected to a SWM 12−5 5V adapter.

The electronic circuitry, detailed in Fig. 3, employs a voltage reference integrated circuit (MAX6018 [25]) to provide a stable reference potential for the 16-bit differential analog-to-digital converter embedded within a K64F microcontroller [26]. Signal measurement for each channel is performed sequentially using a MAX4053 [27] multiplexer. This multiplexer dynamically connects the voltage reference to each terminal, enabling differential voltage measurements between the reference and adjacent electrodes. A simpler alternative for signal measurement could be implemented using a Raspberry Pi microcontroller [28].

Power isolation for the system is achieved via a DC-DC converter (ITX0505SA [29]), connected to a 5V DC adapter (Wall Power Adapter SWM 12−5 [30]). This configuration electrically isolates the power supply of the microcontroller from the USB port and AC ground, protecting against short circuits and preventing excessive load on the host USB port of the computer. Shielded, low-noise coaxial cables are utilized for all interconnections to minimize electromagnetic interference and ensure the integrity of the low-voltage bioelectric signals. The induced voltage in the physiological serum is measured between the RA, LA, and LL terminals without direct connection to the voltage source; instead, measurements leverage the potential gradient established within the conductive medium. Software-based inversion corrects for the reverse polarity of voltages measured due to the central placement of the anode.

**Fig 4:**
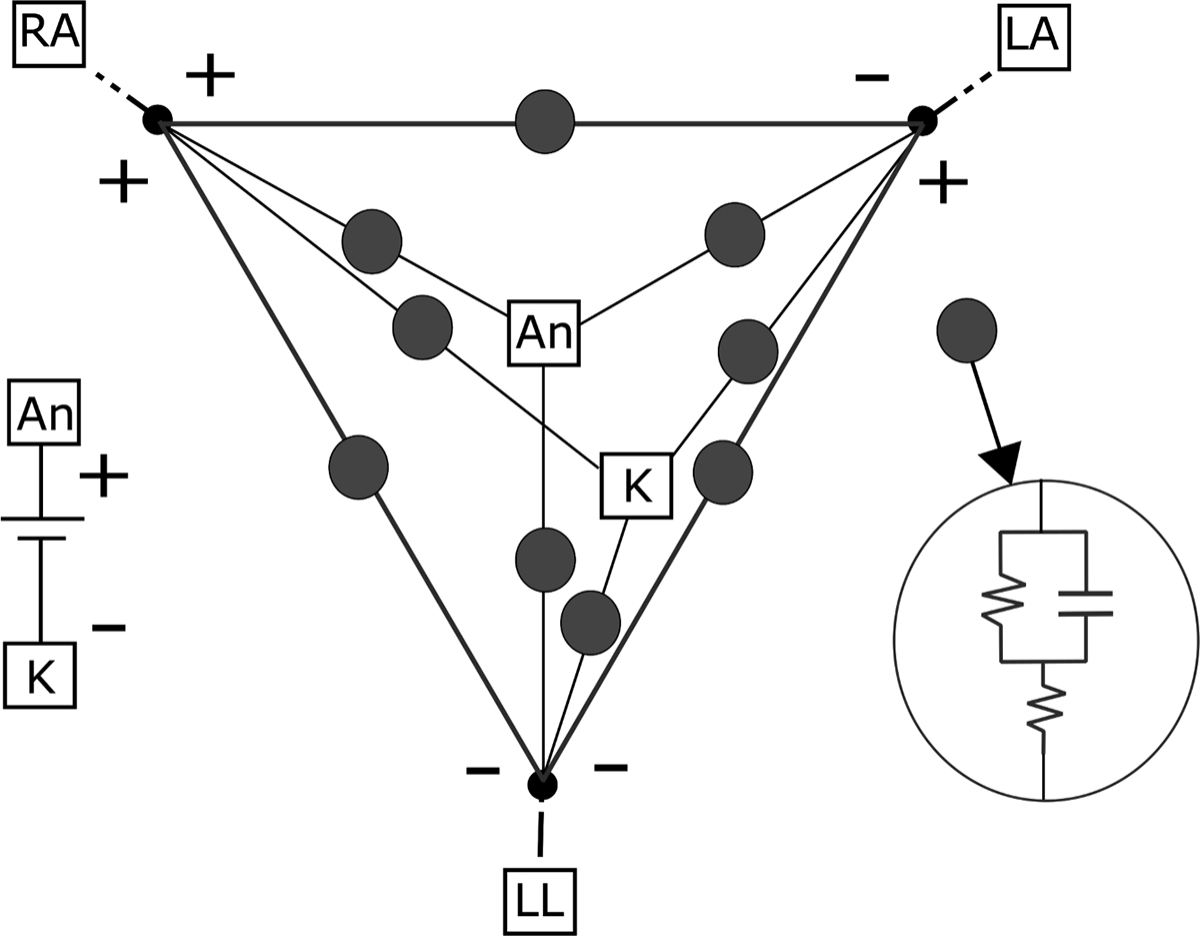
Electrical measurements. Each internodal connection within the conductive medium exhibits electrical impedance behavior that can be approximated by an equivalent circuit consisting of passive elements such as resistors and capacitors.

Electrical impedance behavior of solutions formed by low concentration dilutes can be approximated to a circuit with passive elements such as resistors and capacitors [31],[32] (see Fig 4). At low ionic concentrations, the conductivity concentration relationship is linear and valid for each ion [33]. Although this approach is useful as a first-order approximation, it is important to consider the electrochemical effects that arise due to the interface between the electrodes and the electrolyte solution, as well as the dynamic behavior of the electrical double layer that forms at this interface [22].

Because the anode (An) is the electrode that loses electrons, it experiences the most corrosion among the elements submerged in the conductive medium [23]. To polarize this, we fixed the anode at the center of the container (sacrifice electrode), and designed the cathode (K) to be mobile by connecting it to the oscilloscope probe (mobile hand electrode). This design allows the probe to be cleaned and reused. The cathode, constructed from two metals, can be replaced when corroded, although its design provides enough durability to function for at least four hours without the solution changing color or becoming turbid due to metal particles generated during the experimentation. As the anode electrode is located at the center of the container, the voltages measured by the ADC converters are recorded with reverse polarity compared to the standard representation of the leads. This discrepancy is corrected in the software by inverting the polarity, ensuring it aligns with conventional representation.

#### Software design and data processing.

The Data Processing and Visualization Module is a Python-based [34] application responsible for real-time signal processing, transformation, and graphical representation. The backend of the software acquires the digitized signals representing Leads *DI*, *DII*, and *DIII* from the signal acquisition module. These lead measurements are then analytically processed to calculate the magnitude *V*_*h*_ and angle *α* of the instantaneous heart vector using established analytical geometry principles [22]. Specifically, the following equations are implemented for this transformation (Equation (1):


$$Vh=\frac{DI}{cos\;\alpha };\alpha =tan^{-\text{1}}\left(-\frac{\text{1}}{\sqrt{\text{3}}}-\frac{\text{2}}{\sqrt{\text{3}}}*\frac{DIII}{DI}\right)$$ (1)

The frontend, developed using the Matplotlib library [35], provides a user-friendly interface divided into two main display areas (see Fig 2 right). The left panel presents the temporal waveforms of Leads *DI*, *DII*, and *DIII* allowing for real-time observation of signal amplitudes (ECG waveforms). The right panel concurrently displays the triangle of Einthoven along with the dynamically generated VCG loops, enabling an intuitive visualization of the spatial trajectory of the heart vector. The complete source code, hardware schematics, and raw data used to test the system are open-source and available in the Zenodo repository: https://zenodo.org/records/18486986 (which mirrors the current GitHub development branch: https://github.com/HBprojects/Vectorial-Plot-Rpi-Pico).

#### Technical and educational validation.

The functionality of the VCG prototype was assessed through technical validation and a preliminary educational pilot study to evaluate its pedagogical feasibility.

#### Technical validation.

Was conducted with three methods:

*Method 1: Theoretical Conformity Analysis* The system was prepared following standard operating procedures, including filling the container with physiological serum and calibrating the zero-reference voltage. Users then manually moved the mobile hand-electrode in controlled circular trajectories of varying radius, directions, and angular velocities within the conductive medium. The generated VCG loops and time-domain sinusoidal signals were subsequently analyzed. Conformity was assessed by observing if increasing the radius of the circular path proportionally influenced the amplitude and shape of the time-domain signals, and if changes in angular velocity resulted in proportional temporal variations in the sinusoidal signals.

*Method 2: Real ECG Data Emulation*. To further validate the fidelity of the system, real ECG signals were acquired from the Physionet Database [13], specifically from the Norwegian endurance athlete ECG database v1.0.0 [36]. The digitized DI, DII, and DIII leads data from this database were fed directly into the system bypassing the physical signal acquisition. The VCG loops and ECG waveforms obtained from the VCG simulator were then analyzed to see if their behavior corresponds in terms of shape and time domain to the healthy cardiac conditions of the database.

*Method 3: User-Driven Waveform Generation***.** As a complementary activity, a user was tasked with manually guiding the mobile hand-electrode to reproduce theoretical VCG loops within the container. The objective was to observe if users could intentionally generate characteristic DI, DII, and DIII ECG waveforms through physical interaction. The resulting ECG waveforms were compared using the Dynamic Time Warping (DTW) technique, a signal processing method used to measure similarity between two temporal sequences that may vary in speed [37,38].

#### Educational pilot study.

To assess the feasibility of the prototype for helping users understand concepts related to variations in the position and velocity of the cardiac vector (in both typical and altered cases), and their influence on ECG and VCG tracings, a pilot study was proposed involving advanced medical students and instructors. The study employed a mixed-methods approach combining direct observation and semi-structured interviews. The selected instructors are responsible for teaching cardiac physiology in the medical degree program; two work sessions were conducted with them to familiarize them with the operation of the prototype, and to analyze the objectives, tasks, and learning objectives of the practical activity to be carried out with students. The students were volunteers from the final semesters of medicine, who had already completed basic science courses and acquired knowledge of cardiac physiology using the traditional model (theoretical classes and the analysis of paper-based ECG clinical reports). The research and pilot study received institutional ethics committee approval (Approval Act DVO005 666-CV1026).

The laboratory guide, created with the instructors for the students, includes the following objectives and tasks (see complete guide -English version- in S1 File):

*Learning* objectives:

1. Understand how changes in the magnitude and direction of the cardiac vector relate to the tracings of the bipolar ECG leads and to the VCG loops.
2. Understand how variations in the velocity of the cardiac vector in typical and altered cases influence the ECG and VCG tracings.

*Practice tasks*:

1. Randomly move the VCG hand electrode and analyze its effect on bipolar ECG leads and VCG loops.
2. Reproduce a typical ECG tracing by manipulating the VCG hand electrode.
3. Reproduce an altered ECG tracing (i.e., heart block or other arrhythmias) by manipulating the VCG hand electrode.

At the end of the practical activity, students and instructors were interviewed to provide feedback about the pros and cons of using the prototype and its effectiveness in meeting the proposed learning objectives.

## Results

**Fig 5:**
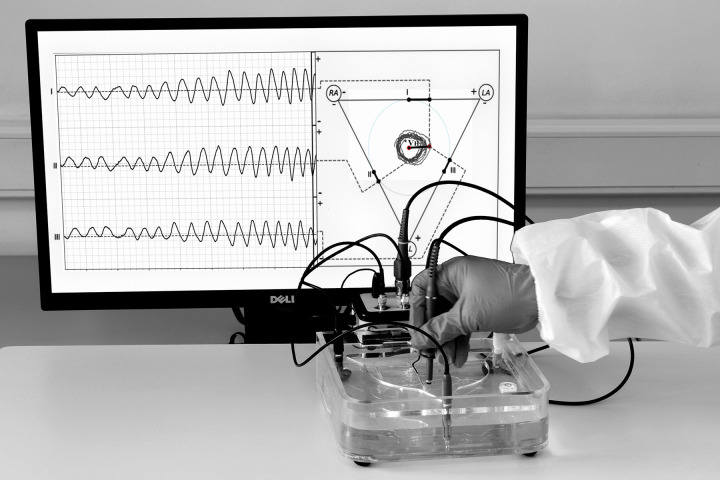
The final vectorcardiography simulator system is shown in operation. According to the technical validation Method 1, here, a user is moving the mobile-hand electrode describing circular trajectories with different radius within the device (see right side of the screen). As expected, the resulting sine waves exhibit different amplitudes in each lead, corresponding to the varying radius of the trajectories (see left side of the screen).

The final VCG simulator system is shown in operation in Fig 5. Here, a user is moving the hand mobile electrode inside the container, describing circular trajectories around the central fixed electrode. The system collects the voltages of leads DI, DII, and DIII and computes the heart vector and its angle in real-time, displaying the results on the screen.

### Preparing the system

To start a work session with the VCG simulator, initial steps and calibration are required. First, it is necessary to connect the cables to the appropriate ports, following the size and color coding. Next, the user should place 400 mL of 0.9% sodium chloride solution (physiological serum) inside the container and verify the horizontal position using the level bubble indicator (E in Fig 2). This amount of solution ensures the electrodes remain submerged to accurately record the electric potential. The user must then check for any potential leaks in the container. If a leak is detected, the instructor should be contacted to replace the device and repeat the process. The following step is to initialize the software interface to establish communication with the device. Finally, a calibration is performed by moving the hand-held electrode to the center of the triangle and pressing the “*Calibration*” button on the interface to establish the zero-reference voltage. Once the setup and calibration are complete, the user can begin interacting with the VCG simulator.

### Technical validation

*Method 1: Theoretical Conformity Analysis*. For this validation method, the container was prepared according to the previous instructions. In this case, the user moves the mobile hand electrode in circular trajectories at various radius, directions, and angular velocities. By analyzing the described VCG loops inside the triangle (right side of the screen in Fig 5), it is observed that as the radius of the circular path increased and changed, the amplitude and shape of the resulting sine waves also changed proportionally (left side Fig 5), following the expected theoretical behavior. Additionally, as the angular velocity of the hand mobile electrode movement changed, the resulting sine waves exhibited proportional changes in the time domain in agreement with the theoretical principles. These validation results confirm the successful implementation of the VCG simulator and its ability to provide a tangible representation of the electrical activity of the heart in real-time.

**Fig 6:**
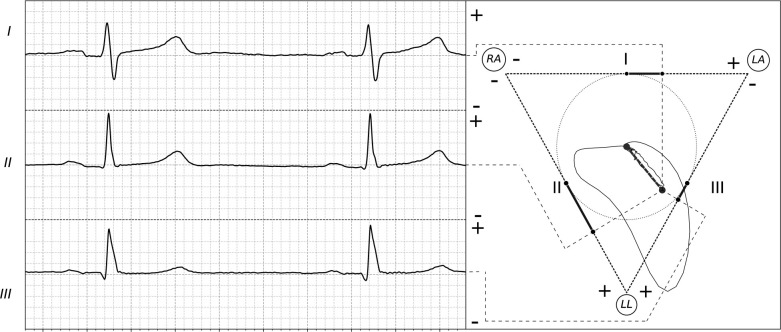
System tested with ECG signal from the Physionet database. The ECG waveforms obtained showed a strong agreement with typical healthy ECG waveforms, both in shape and duration (left). In the case of the VCG loops, the outer VCG loop (corresponding to the QRS complex) created by the system faithfully reproduced the typical “oval-like” shape observed in the literature.

*Method 2: Real ECG Data Emulation*. In this validation method, the system was fed with the Norwegian endurance athlete ECG database. The objective was to verify if the VCG loops and ECG waveforms created by the system matched the expected behavior for healthy heart conditions, in terms of shape and time domain. The results obtained showed a strong agreement with typical healthy ECG waveforms, both in shape and duration (see Fig 6 – left). In the case of the VCG loops, the outer VCG loop (corresponding to the QRS complex) created by the system faithfully reproduced the typical “oval-like” shape observed in the literature, validating the ability of the system to represent the electrical activity of the heart vector (see Fig 6 – right).

**Fig 7:**
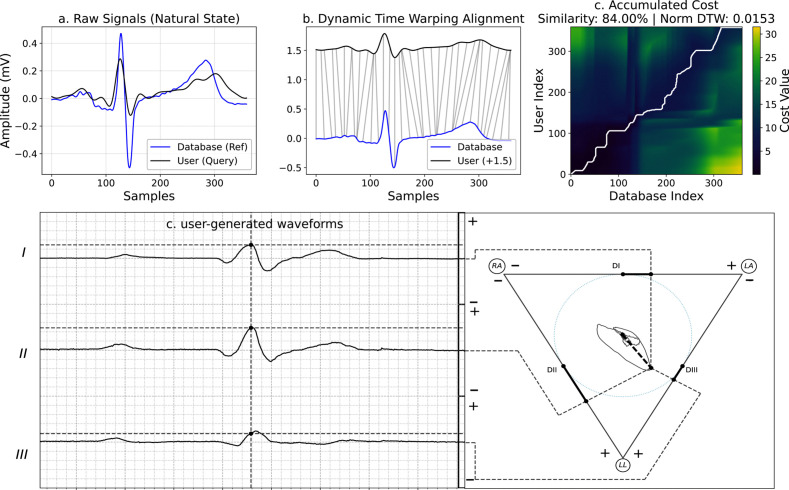
User-generated ECG waveforms and DTW analysis. A user tested the system by moving the hand mobile electrode to create typical ECG waveforms and VCG loops (down). DTW Analysis of User-generated vs. ECG database waveforms (top): **(a)** ECG database reference (blue) and User-generated (gray) waveforms showing differences in amplitude and timing. **(b)** DTW alignment visualization, where gray lines connect corresponding points between the reference and the user signal (shifted vertically for clarity). **(c)** Accumulated cost matrix with the optimal warping path (white line) and the resulting similarity metrics: Similarity 84% and Normalized DTW 0.0153.

*Method 3: User-Driven Waveform Generation.* Here, the system was tested with a user who was asked to create typical DI, DII, and DIII ECG waveforms by moving the hand mobile electrode following the theoretical VCG loops inside the container (see waveforms obtained in Fig 7 – down). The resulting VCG loops and ECG leads displayed the expected shapes. Note the change in the temporal domain between Fig 6 (ECG database waveforms), and Fig 7 – down (User-generated waveforms), since the real ECG signals occur within 70–100 milliseconds, while the hand mobile electrode creates a simulation at a much slower pace (1000–1500 ms). The Dynamic Time Warping analysis compared the two signals, as shown in the top section of Fig 7. The raw signals displayed in (a) panel exhibit morphological similarity but differ in amplitude, with the user signal attenuated and slightly shifted temporally relative to the reference. Panel (b) illustrates the successful alignment achieved by the DTW algorithm, where the gray connecting lines map corresponding morphological features (e.g., peaks and valleys) across the two signals, compensating for the temporal discrepancies. The accumulated cost matrix in panel (c) reveals the optimal warping path (white line), which deviates from the diagonal to minimize the distance between the signals. This alignment resulted in a computed similarity of 84% and a normalized DTW distance of 0.0153, indicating a strong structural correspondence despite the amplitude and timing variations present in the raw data; on the other hand, these quantitative metrics serve as an initial assessment of user-generated waveform fidelity, providing insight into the functional repeatability of the system when manipulated by human users.

Further analysis using the DTW technique on each lead can be found in Supporting Information S1 Fig.

### Educational pilot study

The feasibility and educational potential of the prototype were evaluated through a pilot study involving a total of eight participants: six advanced medical students and two physiology instructors. The assessment employed a mixed-methods approach, utilizing direct observation during the practical sessions and semi-structured interviews upon completion.

*Student Feedback – Usability and Cognitive Integration:* The student cohort, comprised of volunteers in their final semesters of medical school, reported high usability, describing the device as easy to assemble and operate. While the initial correlation between the physical manipulation of the vector and the resulting signal was described as challenging, the structured laboratory guide effectively facilitated a comprehensive learning process. Regarding the specific learning objectives established for the session, the results indicate the following:

*Correlation of Vector Magnitude and Direction:* Validating the objective to understand how vector changes relate to ECG tracings, several participants identified the physical representation of the heart and cardiac vector as a critical component for integrating theoretical concepts. One student highlighted the integrative nature of the activity, stating: *“Veo una ventaja clara en apoyar toda la teoría que vi en el pasado”* (I see a clear advantage in supporting all the theory I saw in the past with this practical activity).

*Influence of Velocity in Altered Cases:* The feedback confirms that the objective regarding the influence of vector velocity in pathological cases was met. After grasping the device mechanics, students reported success in reproducing arrhythmias. One participant noted: *“Fue fácil para mí generar un ECG simulando un bloqueo”* (It was easy for me to generate an ECG simulating a block).

*Critical Thinking:* Some participants emphasized that the activity required active cognitive engagement rather than passive observation. Addressing the cognitive load required to complete the tasks, a student remarked: *“Hay que pensar sí o sí para integrar y comprender”* (One is forced to think to integrate and understand).

A limitation reported by one student regarding the physical design was the restricted space within the triangle setup, which they found constrained their working area during the exercises.

Regarding the Instructor feedback, the two instructors evaluated the potential of the prototype as a teaching aid, particularly praising the interactive component for fostering sensory integration. They noted that the progressive exercises allowed students to adapt quickly to the equipment, facilitating a self-guided learning experience. A key differentiator highlighted by the instructor was the open-ended nature of the simulation compared to traditional tools. As one instructor observed, unlike other teaching devices where work is *“predeterminado y limitado”* (predetermined and limited), this prototype allows for dynamic exploration. Consequently, the instructors concluded that the simulator could facilitate the comprehension of complex electrophysiological parameters (including changes in velocity, polarity, and magnitude) and their clinical integration.

## Discussion

The successful integration of hardware and software components in this study demonstrates the technical feasibility of bridging the gap between theoretical cardiac electrophysiology and practical experimentation. The validation results indicate that the system translates manual vector movements into canonical VCG loops and ECG waveforms, showing strong concordance with both theoretical projections and real physiological data from the PhysioNet database. By enabling users to physically manipulate the dipole source within a conductive medium, the system provides a visual feedback loop that is absent in purely computational simulations. This immediate, experiential engagement creates an educational environment where novice learners can construct mental models of complex cardiac electrical activity through direct interaction.

The technical validation was conducted through three complementary methods, each addressing different functional aspects of the system. The theoretical conformity analysis (Method 1) demonstrated that when circular trajectories of varying radius and angular velocities were manually executed, the resulting VCG loops and sinusoidal signals exhibited proportional changes in amplitude and duration. These results confirm that the system behaves in accordance with theoretical expectations for a moving dipole in a conductive volume, validating the fundamental electro-physical principles underlying the design [22]. In Method 2, the real ECG data emulation, using signals acquired from the PhysioNet database, showed that the system accurately processed and visualized physiological-grade signals. The resulting VCG loops faithfully reproduced the characteristic “oval-like” morphology reported in the literature for healthy cardiac electrical activity [12,22]. This confirms that the signal processing algorithm possesses sufficient fidelity to generate VCG loops consistent with real data, validating the utility of the system not only for simulation but also for the visualization and analysis of existing ECG signals. The user-driven waveform generation (Method 3), where participants attempted to manually reproduce typical ECG waveforms by manipulating the mobile electrode, provided further evidence of the interactive capabilities of the system. The DTW analysis revealed a significant correlation between the user-generated waveforms and the reference physiological signals, despite the inherent time-scale differences [37,38]. This finding underscores the effectiveness of the simulator in allowing users to intuitively construct physiologically relevant cardiac electrical activity representations through physical interaction.

The preliminary educational validation demonstrates the pedagogical feasibility of the simulator and reveals a crucial aspect of its teaching value. Although the container represents a geometric simplification of the human thorax (not homogeneous) and does not take into account the off-center position of the heart (an anatomical reality that influences VCG loop morphology [20]), the capacity of the system to produce VCG loops and ECG signal tracings consistent with established literature validates the educational efficacy of this simplified model. A notable difference in operation is the temporal scale: manual simulation is orders of magnitude slower than real cardiac cycles, which occur within 70–100 milliseconds. Rather than a limitation, this “slow-motion” generation proves advantageous from a pedagogical perspective. Research in experiential learning theory posits that knowledge is constructed through reflection on experience, and that optimal learning occurs when each phase of the learning cycle (concrete experience, reflective observation, abstract conceptualization, and active experimentation) is experienced fully [39,40]. By slowing down the rapid cardiac cycle, the simulator allows students to deconstruct the electrical activity phase by phase (P wave, QRS complex, T wave), providing the necessary temporal space for reflection and comprehension. This aligns with simulation-based learning principles, which have been shown to enhance the self-confidence of the students and combine various forms of knowledge into a coherent conceptual framework [41].

Moreover, the student and instructor feedback from the pilot study provides empirical support linking these theoretical learning principles with practical application. Students reported that the activity required active cognitive engagement, noting that they “had to think to integrate and understand,” which is precisely the type of critical thinking that experiential learning seeks to foster [39]. The instructors particularly valued the open-ended nature of the exploration, contrasting it with traditional devices where work is “predetermined and limited.” This flexibility is consistent with Kolb’s learning cycle, which emphasizes active experimentation as a crucial component of knowledge construction [42]. The positive feedback on the use of open-source software is also significant, as it facilitates adoption in resource-constrained educational settings and encourages customization by educators. The ability to modify and extend the software aligns with constructivist learning principles, which posit that learning is an active process of knowledge construction rather than passive reception [42,43].

When critically compared with existing educational approaches in the literature, our simulator offers distinct pedagogical advantages. Jin et al.‘s Einthoven triangle device provides a valuable static model for understanding lead geometry, enabling students to physically interact with a representation of cardiac electrical activity and measure lead voltage values [15]. However, this device lacks dynamic visualization capabilities and cannot display the temporal evolution of the heart vector through P, QRS, and T loops. The incorporation of real-time software visualization in the simulator proposed here addresses this gap by allowing the observation of these temporal dynamics. On the other hand, software-based applications, such as the multimedia educational application proposed by Šljivo et al. [14] or the BRAVEHEART Matlab package [12], provide sophisticated visualization and analysis capabilities but rely predominantly on pre-recorded data and virtual interactions. While these tools offer powerful analytical features, their purely computational nature lacks the crucial physical interactivity in line with active learning theories [4,5]. Unlike pure software simulations or the interpretation of paper-based ECG reports, the physical component of the system proposed here introduces proprioceptive feedback (the sense of body position and movement) which can significantly reinforce spatial learning. Educational research provides strong support for this multimodal approach: tactile manipulation of physical models has been shown to help learners overcome the high cognitive load associated with mental rotations and enhance spatial understanding [44,45]. The physical interaction of moving the hand electrode manually in our system thus provides a multisensory learning experience that engages multiple cognitive pathways, facilitating the integration of theoretical concepts with spatial understanding in ways purely visual software cannot.

Despite its innovative features and demonstrated potential, several limitations of the current prototype should be acknowledged. From a technical standpoint, electrode corrosion during long-term use represents a practical limitation that may impact system durability and reliability in high-throughput educational settings. While this is mitigated in our design by appropriate metal combinations for the sacrificial anode and the configuration allowing for electrode replacement, it could be further reduced by optimizing electrode materials through more extensive electrochemical characterization [23]. Furthermore, while the technical validation established the conformity of the system to theoretical principles and its ability to reproduce physiological signals, a comprehensive characterization of specific performance metrics such as resolution, repeatability (beyond user-driven fidelity), signal drift, and an exhaustive analysis of inter-user variability has not yet been performed. The current iteration focused primarily on demonstrating pedagogical feasibility and functional proof-of-concept, rather than an engineering-grade performance evaluation. However, the Dynamic Time Warping analysis in Method 3 provides an initial quantitative assessment of user-generated waveform fidelity, which indirectly addresses aspects of functional repeatability and inter-user consistency in reproducing target waveforms.

Regarding the representation of electrical heart vector activity using a homogeneous saline solution, it is important to clarify that the system presents a simplified model of actual physiological conditions. The human thorax is a non-homogeneous conductor with complex anisotropic conductivities, and the heart is not positioned at the center of the lead system. These anatomical factors influence VCG loop morphology in clinical recordings; thus, the simplified model should be presented to students as such to prevent misconceptions about real-world variability [20]. Additionally, one student noted that the restricted interaction space within the triangular setup constrained their working area during exercises; expanding the geometric dimensions of the conductive medium would improve usability and allow for more expressive vector manipulations.

The findings of this study carry several key take-home messages for the medical and bioengineering education communities. First, this VCG simulator successfully demonstrates that complex cardiac electrophysiology concepts can be made tangible and manipulable through low-cost hardware and open-source software. The system democratizes access to advanced VCG training tools by using inexpensive materials and replicating complex bioelectrical phenomena without requiring expensive clinical equipment. Second, pilot study data confirm the benefits of active experiential learning. Students and instructors reported greater cognitive engagement and exploration opportunities with the simulator than with traditional methods. This aligns with simulation-based education research highlighting interactive learning for clinical reasoning [41,46]. Third, the flexibility of the system supports diverse educational goals: from basic Einthoven triangle principles to advanced topics like arrhythmia simulation and lead theory. Instructors noted its support for “dynamic exploration” over fixed exercises, fostering self-directed learning key to clinical problem-solving. As a scalable yet sophisticated tool for resource-limited labs, it promotes vectorcardiography in medical curricula and improves future clinical grasp of cardiac electrical activity.

### Future work

Considering the limitations mentioned above, future work will include a detailed engineering characterization of the performance of the system, focusing on quantitative metrics such as signal resolution, measurement repeatability under controlled conditions, long-term drift, and a more extensive statistical analysis of inter-user variability during practical tasks. Additionally, efforts will be directed towards developing advanced signal processing tools for clinical features like QRS detection, ST-segment detection, as well as heart rate variability analysis. Additional functionalities may include software functions that can simulate medication administration effects. Augmenting the simulation with the ability to consider and modify physiological conditions, heart anatomy, and electrode placement would be another useful step.

## Conclusion

This study demonstrates the feasibility and educational value of a low-cost, real-time VCG simulator. Specifically, it integrates physical hardware interaction with digital signal processing to enable immersive experiential learning. Moreover, technical validation confirmed the accurate conversion of manual vector movements into standard VCG loops and ECG waveforms, with results matching theoretical principles and literature descriptions of healthy cardiac morphologies. In addition, Dynamic Time Warping analysis showed strong correlations between user-generated waveforms and reference physiological signals, thereby supporting the ability of the simulator to produce realistic cardiac electrical representations via physical interaction. Complementing these technical achievements, a pilot study with medical students and instructors provided empirical evidence of enhanced cognitive engagement and concept integration; students reported requiring critical thinking for comprehension, while instructors valued its dynamic exploration beyond fixed exercises. However, limitations include electrode corrosion and a simplified conductive medium model. Despite these challenges, the educational benefits of the system, cost-effectiveness with open-source software, and fit with experiential learning make it as a scalable tool for resource-limited cardiac electrophysiology education, ultimately promoting VCG adoption and clinical diagnostics.

## Supporting information

S1 Fig Multi-lead DTW analysis. The left panels illustrate the raw signal superposition for Leads I, II, and III, highlighting significant temporal phase shifts between the Real (User-generated -black-) and Synthetic (ECG database -colored-) datasets. The right panels display the waveforms after applying elastic time shifting, aimed at aligning the P-wave, QRS complex, and T-wave features. The resulting Warped Correlation coefficients (ρ > 0.92 across all leads) quantify the morphological similarity, with Lead I exhibiting the highest fidelity (ρ = 0.950). These metrics validate the ability of the system to accurately reproduce cardiac vector projections despite temporal variability.
(TIFF)

S1 File Laboratory Guide. A comprehensive step-by-step manual for students. This document includes the experimental setup protocols, safety instructions, and a set of practice tasks designed to reinforce the concepts of vectorcardiography using the simulator.
(PDF)
