# Supplementary material for: Real-time vectorcardiography simulator system
Source: PLoS One. 2026 Mar 31;21(3):e0345612. doi: 10.1371/journal.pone.0345612 (PMC13037978; doi:10.1371/journal.pone.0345612)
Supplement: S1 File — A comprehensive step-by-step manual for students. This document includes the experimental setup protocols, safety instructions, and a set of practice tasks designed to reinforce the concepts of vectorcardiography using the simulator. (PDF) [file pone.0345612.s002.pdf]

THE CARDIAC VECTOR AND ECG CORRELATION

A Laboratory Guide for the Real-Time Vectorcardiography System

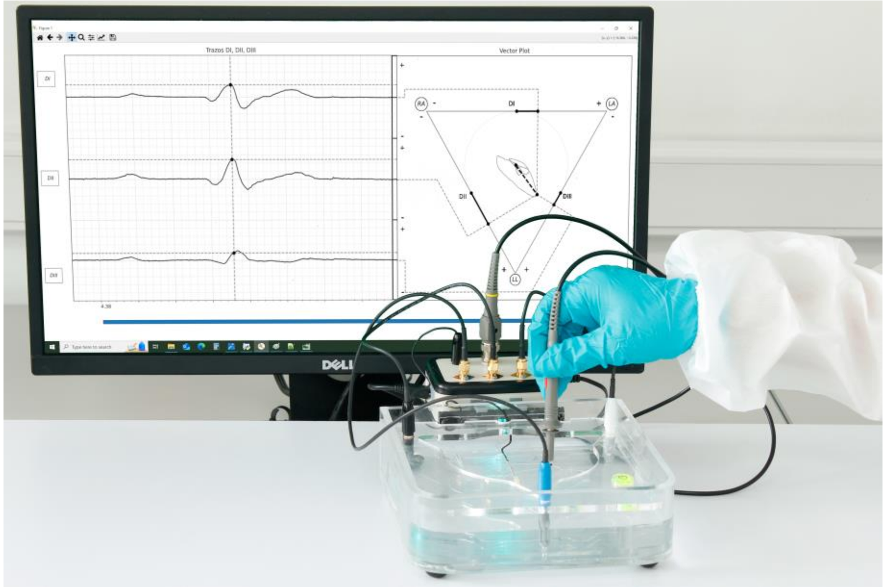

Fig. 1. Vectorcardiography simulator system assembled.

INTRODUCTION

The purpose of this laboratory is to understand the formation of the cardiac vector as a phenomenon of electrical conduction through a hands-on practical session. Cardiac electrical activity is a spatial phenomenon whose interpretation requires a deep understanding of the cardiac dipole. The conventional electrocardiogram (ECG), although it remains a highly useful clinical tool, should be understood as a limited projection of a three-dimensional vector onto a single scalar axis, which inherently entails a loss of critical data. Vectorcardiography (VCG) overcomes this limitation by capturing the cardiac electrical vector. The transition from scalar to vector analysis allows the clinician to explicitly observe the relationships among Leads. While the ECG fragments the signal, VCG integrates magnitude and direction over time, making it possible to identify spatial disorganization of electrical forces before these manifest as voltage changes on traditional thermal paper recordings. This approach simultaneously enables a more accurate interpretation of electrocardiographic tracing events. In this experimental session, a manual simulation of a tracing will be performed using a container filled with saline solution (see Fig. 1). When connected to an electronic signal acquisition device, this setup allows correlation between the changes made within the container and the resulting waveforms, thereby simulating leads DI, DII, and DIII under normal conditions as well as in scenarios involving cardiac abnormalities.

LEARNING OBJECTIVES

- Understand how changes in the magnitude and direction of the cardiac vector relate to the tracings of the bipolar ECG Leads and to the VCG loops.
- Understand how variations in the velocity of the cardiac vector in typical and altered cases influence the ECG and VCG tracings.

THEORETICAL FRAMEWORK

The graphic representation of cardiac electrical activity (typical ECG) is the macroscopic manifestation of a perfectly orchestrated ionic choreography. For the health sciences student, the challenge lies in translating the microscopic activity of cellular membranes into a three-dimensional vector representation on the human torso.

The cellular basis of synchrony

The heart's mechanical efficiency depends on its ability to act as a functional syncytium. This coordination would be impossible if cardiomyocytes were isolated entities. The myocardial architecture is integrated by low-resistance structures called gap junctions, which act as "ionic bridges." The movement of ions between the intracellular and extracellular spaces during the depolarization and repolarization of cardiac cells propagates thanks to this low impedance. This is vital for the impulse to spread as a continuous wave, allowing billions of cells to activate in synchrony and transforming the tissue into a single functional unit.

The electrical dipole and conduction

This cellular infrastructure is the biological foundation that allows for the electrical dipole a progressive electrical change across the cardiac fibers (see Fig. 2).

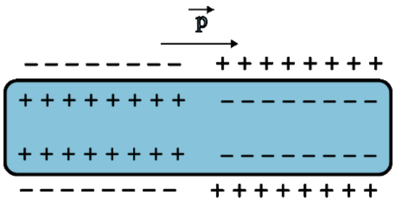

Fig. 2. Representation of a dipole.

The impulse originates in the natural pacemaker (SA node) and propagates radially through the atrial walls to the atrioventricular (AV) node. Here, the impulse P encounters a zone of very slow conduction. This delay in potential transmission allows atrial systole to conclude and the ventricles to reach optimal filling before ventricular contraction. Once past the AV node, the velocity increases drastically. The interventricular septum depolarizes, and the impulse travels in parallel through the ventricular fascicles. Due to the mass of the left ventricle, its activation is more prolonged than that of the right.

Vector modeling and Einthoven's Law

This orderly movement of charges generates a dynamic electrical field that can be physically modeled via Vectorcardiography or the ECG [1] (Fig. 3).

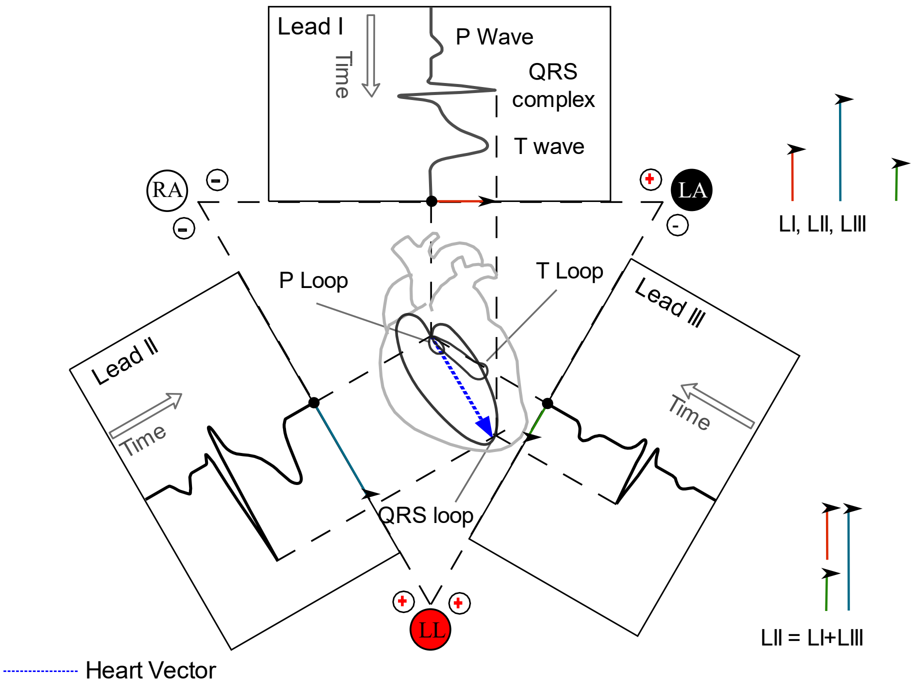

Fig. 3. Vector representation of the heart's electrical activity.

Einthoven was the first to record the heart's electrical activity by considering the thorax as an equilateral triangle (Fig. 4 - right) with the heart positioned at the center. He imagined that the electrical state of the heart could be represented by a single vector drawn from the center of the triangle, using the projections of the vectors traced along its sides. However, a more precisely anatomical way to represent this phenomenon is using a scalene triangle, as in Fig. 4 -left [2].

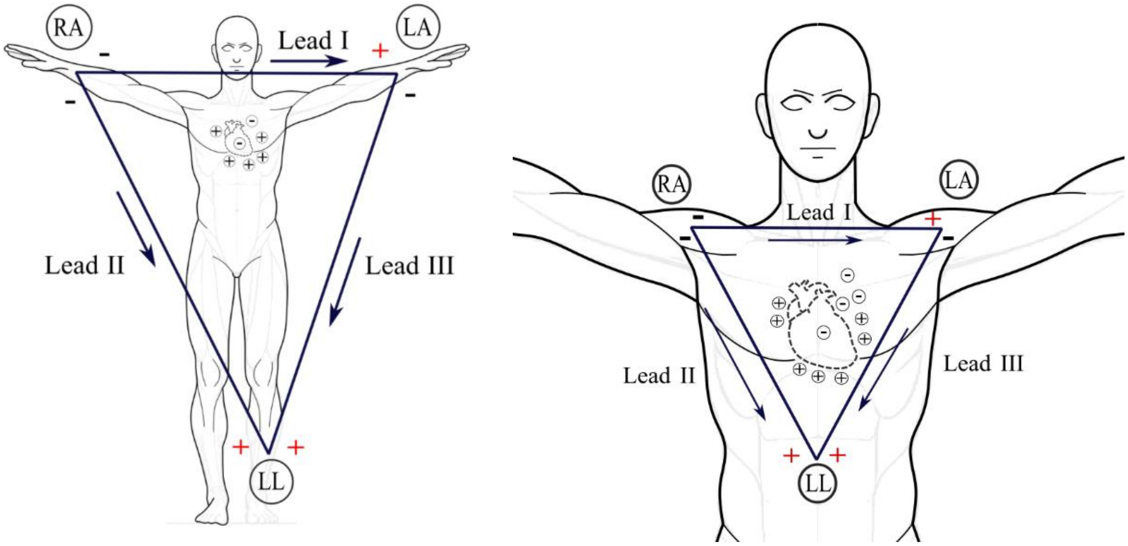

Fig. 4. Two representations of the heart location relative to the electrode’s distribution; Left: The scalene triangle, Right: Einthoven triangle.

ASSEMBLY PROCEDURE

Materials per group

Verify that the following items are available in the work area (see Fig. 5 as a reference):

- Electronic module
- Oscilloscope probe 1:1
- Peripheral electrodes
- Power supply
- USB cable
- 500 mL of saline solution (0.9% NaCl) or Ringer's lactate
- Fixed electrode (anode)
- Container
- Vectorcardiograph software

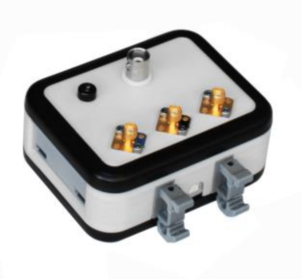 Electronic module	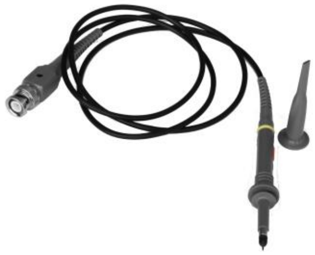 Oscilloscope probe 1:1(Cathode)	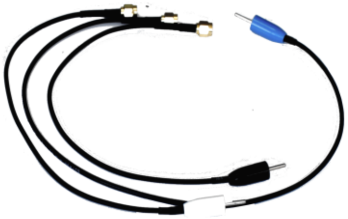 Peripheral electrodes
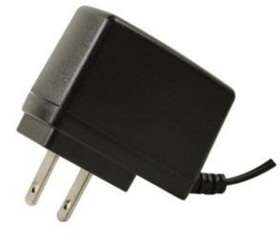 Power supply	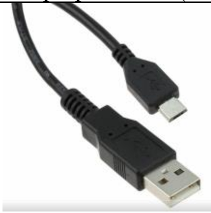 USB cable.	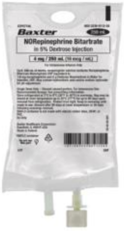 500 mL of saline solution (0.9% NaCl) or Ringer's lactate
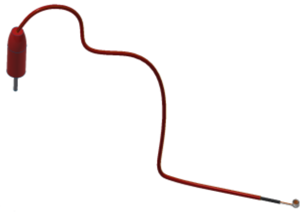 Fixed electrode (anode)	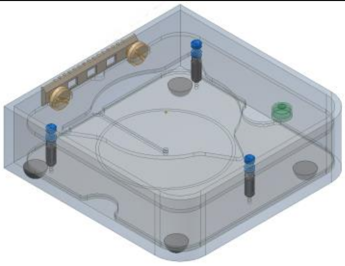 Container	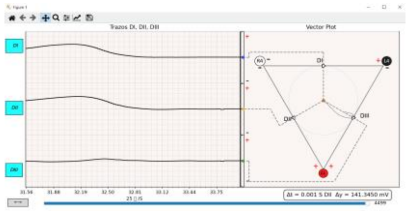 Software.

Fig. 5. Required materials.

Device assembly

Verify that the previously identified components are connected as indicated below. If this is not the case, arrange them according to the following graphic guide: from Fig. 6 to Fig. 9.

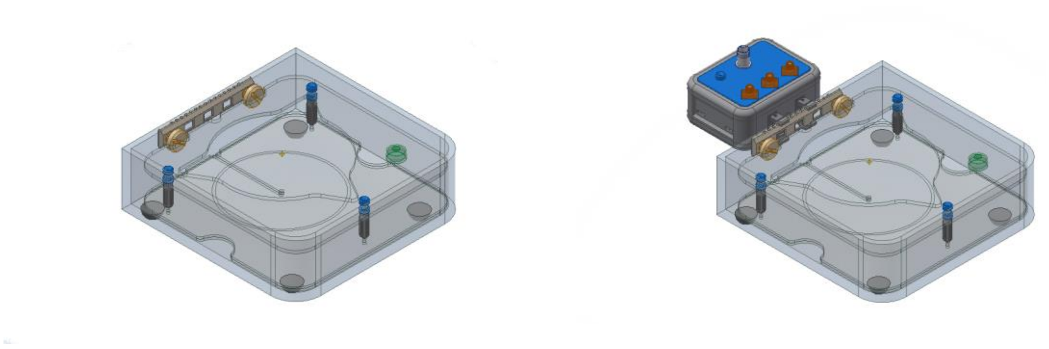

Fig. 6. Assembly of the electronic module to the container.

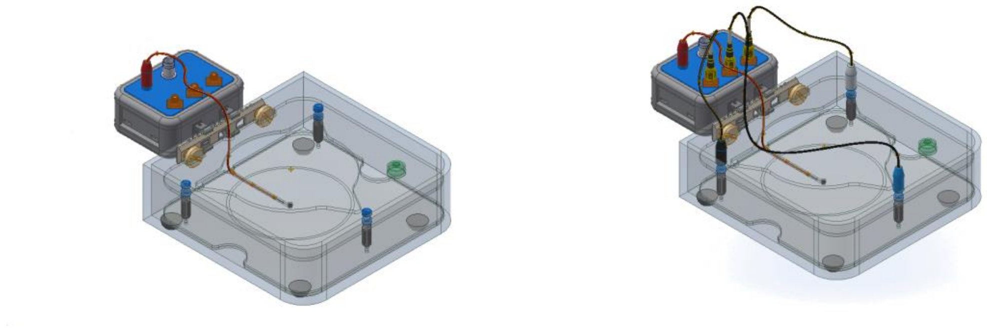

Fig. 7. Connection of the reference electrode at the center of the container and connection of the peripheral electrodes.

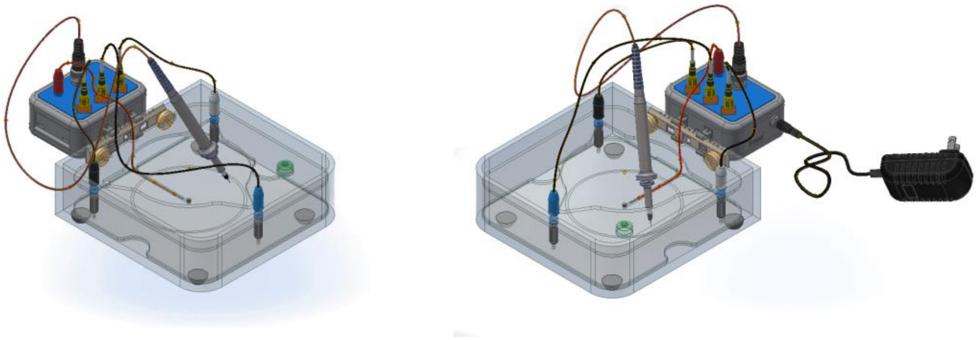

Fig. 8. Connection of the probe and voltage supply.

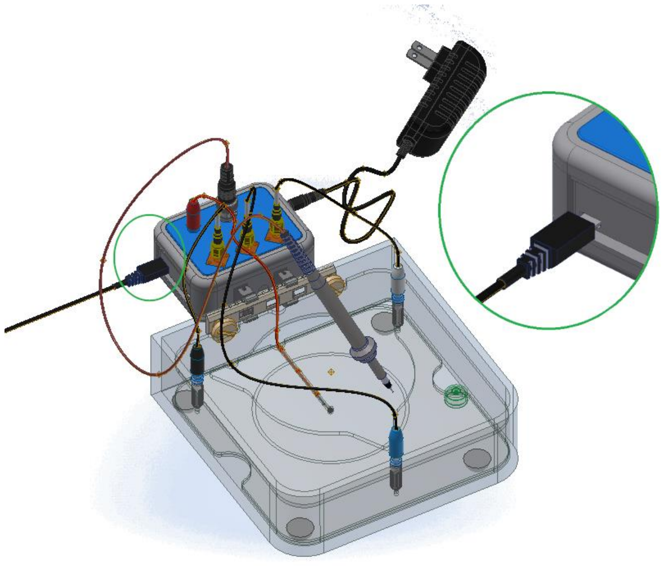

Fig. 9. Connection of the USB cable.

Important software operation notes

Verification of electrode connections
If all electrodes are properly connected and have good contact, an aqua-colored light will turn on in the electronic module, indicating proper connection. If the light is white, check the connections and wait a few seconds for the device to verify them. Once the connections are correct, the light color will change.

Set zero reference (baseline adjustment)
Due to small residual electrical charges on the surrounding electric field, it is normal for the zero reference to drift or not appear centered. To correct this displacement, place the probe tip near the center of the container, then press the "z" key on the keyboard and wait two seconds.

Pausing the system
At any time, the system can be paused by pressing the "p" key on the keyboard. The arrow keys allow navigation through the tracing captured up to that point. The mouse enables correlation between points in the tracing and the vectorcardiogram.

Activation of the test signal
The device includes a pre-recorded electrocardiographic signal that is generated as if the probe were being moved inside the container. This signal can be activated by pressing the "d" key on the keyboard.

Reduction of tracing speed
The number of data points displayed on the screen can be modified by pressing the "m" key on the keyboard. The tracing updates automatically, preventing screen saturation. This function allows switching between four tracing speeds by pressing the key successively; pressing it a fifth time returns the system to the initial speed.

TASKS

Task 1. Randomly move the VCG hand electrode and analyze its effect on bipolar ECG leads and VCG loops

Familiarization with the device, axis orientation, and electrode placement

- Once the zero reference has been set, insert the probe into the container with the saline solution and observe the hand movement and its corresponding representation on the screen during each tracing.
- Each time a tracing is performed, return the probe to the point designated as zero in the container to reestablish the baseline on the screen graph. The tracings produced by moving the hand inside the container should show a certain degree of correlation with those displayed on the screen, and it is important to verify the signal polarity. For example, if the hand moves from the center toward the right, there should be agreement with other tracings in the same direction.
- Attempt to generate completely positive waves in each lead, and identify the positive and negative sides of each Leads (DI, DII, and DIII).

Understanding the difference between amplitude and frequency

- Set the zero reference. Move your hand while drawing circles with the tip of the oscilloscope probe around this point (see Fig. 10). Keep the zero point at the center of each circle you draw. Make two or three circles, then perform additional circular movements while increasing the diameter. Try to maintain the same movement speed at all times. Observe and verify the changes that appear in the tracing.
- Position the oscilloscope probe at a moderate distance from the zero reference and draw concentric circles. Keep the circles the same size while varying the speed of the movement, and monitor the resulting changes in the tracing.
- The previous exercises demonstrate changes in the tracings related to amplitude and frequency. This correlation supports the understanding of what occurs in an electrocardiogram when heart rate increases or decreases (when you move your hand faster or slower). It also allows visualization of the impact of changes in vector magnitude when larger circles are drawn, analogous, for example, to what occurs during hypertrophy, when the electrical contribution per cell is greater than in normal conditions.

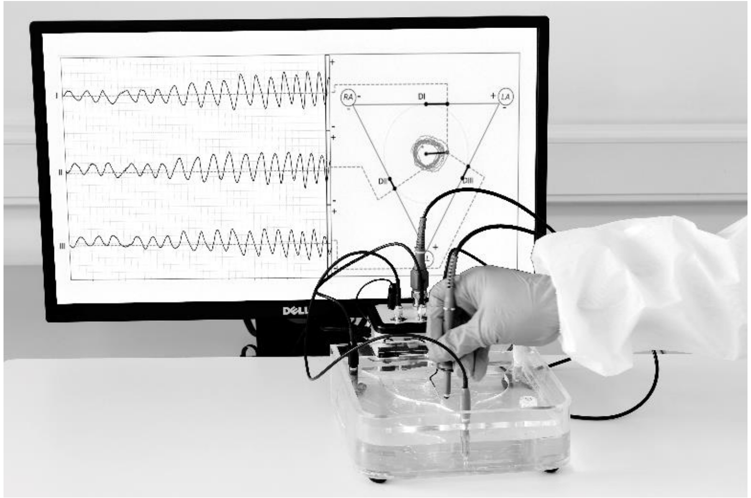

Fig. 10. Understanding the difference between amplitude and frequency.

Task 2. Reproduce a typical ECG tracing by manipulating the VCG hand electrode.

Formation of an ECG Signal

- Now attempt to generate waves resembling a P wave and a QRS complex, considering the magnitude and direction of the vector in each case. We recommend reviewing the test signal at this stage by pressing the “d” key. This signal will serve as an example and guide for the procedure.
- While carrying out the exercise, identify the difficulties encountered in achieving the desired waveforms, and discuss them with your classmates or with the instructor.
- Attempt to reproduce a QRS complex with a normal axis, as well as right-axis deviation and left-axis deviation, and examine the changes that these directional variations produce in leads DI, DII, and DIII on the ECG.
- Attach to the lab report a screenshot showing a normal tracing (see Fig. 11 as reference).

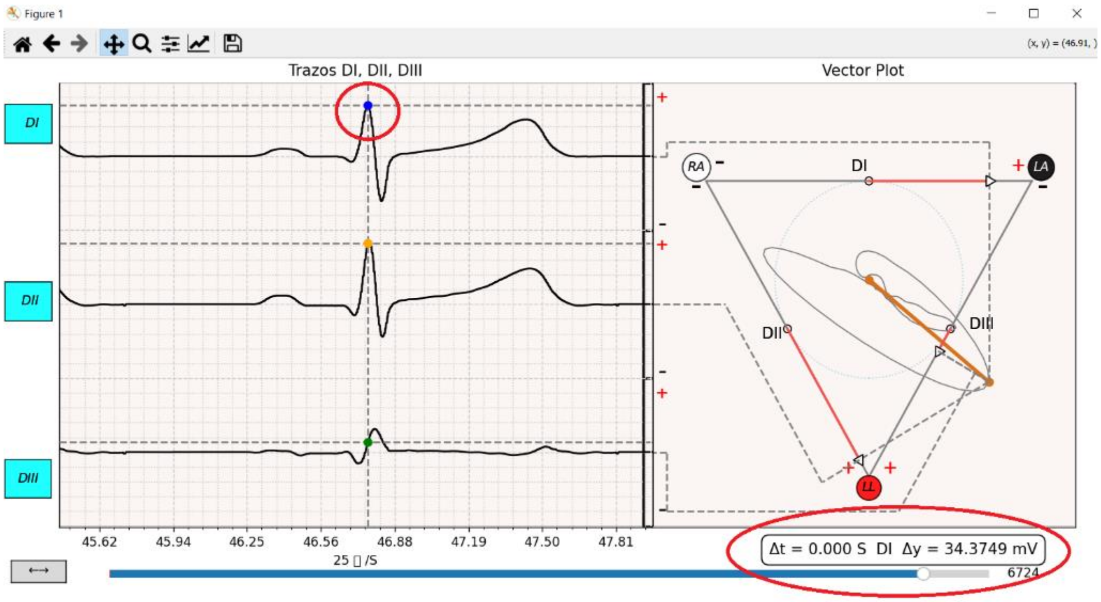

Fig. 11. Normal ECG waveforms and VCG loops.

Task 3. Reproduce an altered ECG tracing (i.e. heart block or other arrhythmias) by manipulating the VCG hand electrode.

- Repeat the steps in Activity 2, but now try to generate tracings similar to those observed in arrhythmias or rate abnormalities. For example, generate loops that produce broader and wider complexes, or more frequent complexes with normal or abnormal morphology, as observed in conditions such as hypertrophy, conduction blocks, and monomorphic or polymorphic tachycardias, respectively. Attach to the lab report screenshots showing these abnormal tracings.

- Discuss all the observations (Tasks 1 to 3) with your classmates and the instructor, and clearly state the conclusions of the laboratory practice. Document your findings in a lab report including the graphical records captured during the procedures.

READINGS

Sumche Man, S., Maan, A. C., Schalij, M. J., & Swenne, C. A. (2015). Vectorcardiographic diagnostic and prognostic information derived from the 12-lead electrocardiogram: Historical review and clinical perspective. *Journal of Electrocardiology, 48*(4), 463–475.
This review article presents a comprehensive historical overview and clinical evaluation of vectorcardiography derived from the standard 12-lead ECG. The authors discuss the mathematical and physiological principles underlying vector reconstruction, as well as the diagnostic and prognostic value of vector-based analysis in clinical cardiology. This source directly supports the vectorial analysis, providing clinical context for concepts such as cardiac axis deviation, spatial depolarization patterns, and rhythm abnormalities explored experimentally in the practice.

Feher, J. J. (2012). *Quantitative Human Physiology*. Academic Press – Elsevier. ISBN: 978-0-12-382163-8, Chapter 5.6.
This textbook provides a quantitative and systems-based approach to human physiology, with detailed treatment of bioelectric phenomena in excitable tissues. The sections on cardiac electrophysiology describe the generation and propagation of electrical potentials in the heart, forming the theoretical basis for understanding the cardiac dipole and vector formation. This supports the foundational concepts of ionic displacement, electrical conduction in biological media, and the interpretation of electrocardiographic signals from a biophysical perspective. It is particularly useful for linking physiological mechanisms to measurable electrical signals.
